# Targeting Oxidative Stress and Inflammation in Pembrolizumab-Induced Renal Injury: A Comparative Evaluation of the Protective Effects of Flunarizine and Carvacrol in Rats

**DOI:** 10.3390/biom16060786

**Published:** 2026-05-27

**Authors:** Engin Hendem, Bulent Yavuzer, Esra Tuba Sezgin, Murat Gunay, Mustafa Ozkaraca, Ali Gungor, Durdu Altuner, Halis Suleyman

**Affiliations:** 1 Department of Medical Oncology, Mengucek Gazi Education and Research Hospital, Erzincan Binali Yıldırım University, Erzincan 24100, Turkey; engin.hendem@saglik.gov.tr; 2 Department of Pharmacology, Faculty of Medicine, Erzincan Binali Yıldırım University, Erzincan 24100, Turkey; bulent.yavuzer@erzincan.edu.tr (B.Y.); daltuner@erzincan.edu.tr (D.A.); 3 Anesthesia Program, Vocational School of Health Services, Erzincan Binali Yıldırım University, Erzincan 24036, Turkey; esra.demir@erzincan.edu.tr; 4 Biochemistry Laboratory, Mengucek Gazi Education and Research Hospital, Erzincan Binali Yıldırım University, Erzincan 24100, Turkey; mgunay@erzincan.edu.tr; 5 Department of Pathology, Faculty of Veterinary Medicine, Sivas Cumhuriyet University, Sivas 58140, Turkey; mustafaozkaraca@cumhuriyet.edu.tr; 6 Laboratory and Veterinary Health Program, Vocational School of Health Services, Osmaniye Korkut Ata University, Osmaniye 80000, Turkey; aligungor@osmaniye.edu.tr

**Keywords:** 3,3′-dityrosine, carvacrol, cyclooxygenase-1/cyclooxygenase-2, flunarizine, hepatitis A virus cellular receptor 1, immune checkpoint inhibitors, double immunofluorescence, nephrotoxicity, oxidative stress, pembrolizumab

## Abstract

Background: Pembrolizumab, a programmed cell death protein 1 (PD-1) inhibitor, is widely employed in oncological practice; however, its propensity to induce nephrotoxicity through immune-mediated oxidative and inflammatory mechanisms remains an insufficiently characterized clinical concern. The present study comparatively investigated the renoprotective effects of flunarizine, a voltage-dependent calcium channel antagonist, and carvacrol, a monoterpene, against pembrolizumab-induced renal injury in rats. Methods: Twenty-four male Wistar albino rats were assigned to four groups (*n* = 6): healthy control (HG), pembrolizumab alone (PZB), flunarizine+pembrolizumab (FLPZ), and carvacrol+pembrolizumab (CCPZ). Pembrolizumab was administered intraperitoneally at 5 mg/kg; flunarizine orally at 5 mg/kg and carvacrol intraperitoneally at 50 mg/kg, once daily for seven consecutive days. Renal oxidative status was assessed by measuring malondialdehyde (MDA) and total glutathione (tGSH) levels. Histopathological evaluation was performed using hematoxylin and eosin staining. Two double immunofluorescence panels were employed to assess 3,3′-dityrosine/Hepatitis A virus cellular receptor 1 (HAVCR1) and cyclooxygenase-1 (COX-1)/cyclooxygenase-2 (COX-2) expression, respectively. Results: Pembrolizumab caused pronounced oxidative stress and inflammatory responses in renal tissue, leading to a significant increase in renal MDA levels and a marked decrease in tGSH levels. These biochemical alterations were accompanied by severe tubular degeneration and increased expression of 3,3′-dityrosine, which is associated with oxidative damage, as well as HAVCR1, a marker of cellular injury, and COX-1 and COX-2, which reflect inflammatory activity. These findings indicate that pembrolizumab disrupts the renal redox balance and activates both oxidative and inflammatory pathways in kidney tissue. Flunarizine and carvacrol significantly reduced these pathological changes. Both agents attenuated oxidative stress markers and supported antioxidant defenses, thereby alleviating tissue damage. However, flunarizine demonstrated a more pronounced renoprotective effect across all evaluated parameters, restoring MDA and tGSH levels closer to physiological values and reducing tubular injury to a minimal level. Carvacrol showed a more limited but still statistically significant protective effect. Conclusions: Both agents confer significant renoprotection against pembrolizumab-induced oxidative injury; however, flunarizine exhibits a more robust protective profile, likely attributable to its capacity to attenuate calcium-mediated mitochondrial dysfunction and preserve cellular bioenergetic homeostasis.

## 1. Introduction

Recent years have witnessed significant advances in cancer treatment driven by immunotherapy. In particular, the blockade of immune checkpoint pathways, such as cytotoxic T-lymphocyte antigen-4 (CTLA-4) and programmed cell death protein 1 (PD-1), through the use of monoclonal antibodies has demonstrated promising therapeutic outcomes in advanced-stage cancers [1,2]. In this context, pembrolizumab, a PD-1 inhibitor, is an immune checkpoint inhibitor (ICI) approved for the treatment of various solid tumors and hematological malignancies. It is currently preferred, particularly in the treatment of non-small-cell lung cancer (NSCLC) [3] and head and neck squamous cell carcinoma (HNSCC) [4]. However, immune-related adverse events resulting from excessive immune activation can limit the clinical use of these agents [5]. Although relatively uncommon, renal injury is considered a clinically significant adverse effect of these agents [6]. Nephrotoxicity that may develop due to antineoplastic agents is a condition that leads to oxidative stress as a result of increased production of reactive oxygen species (ROS). When these free radicals are not adequately neutralized, lipid peroxidation (LPO) and impairment of antioxidant defense mechanisms may develop [7]. ICIs activate T cells by blocking the PD-1 signaling pathway, thereby enhancing antitumor immune responses. However, this immune activation may also contribute to renal injury through autoimmune responses against renal antigens, reactivation of drug-specific T cells, and cytokine-mediated inflammatory processes. Pembrolizumab-associated renal toxicity most commonly manifests as acute interstitial nephritis and is characterized by inflammatory cell infiltration [8]. Accumulating evidence indicates that pembrolizumab may enhance immune activation in certain tissues, leading to increased proinflammatory cytokine production, elevated oxidative stress, and subsequent inflammatory responses [9]. In addition to these mechanisms, oxidative stress- and inflammation-mediated activation of the renin–angiotensin system (RAS) together with dysregulation of key signaling pathways such as Wnt1/β-catenin may further aggravate podocyte injury and contribute to progressive renal damage [10]. Consistent with the mechanisms underlying renal fibrosis, which is driven by persistent inflammation and oxidative stress leading to progressive structural kidney damage, these pathways may represent a shared pathogenic axis between chronic renal injury and immune-related nephrotoxicity [11]. Recent studies also suggest that pembrolizumab may be associated with metabolic disturbances, including hypercalcemia [12]. As is well established, elevated intracellular calcium (Ca^2+^) can induce mitochondrial overload, leading to ATP depletion [13,14]. A reduction in intracellular ATP production leads to increased ROS generation, thereby inducing oxidative stress [15]. Collectively, these findings suggest that maintaining intracellular ATP and Ca^2+^ homeostasis may be beneficial in the management of pembrolizumab-induced toxicity.

Flunarizine, the drug investigated in this study to evaluate its potential protective effects against pembrolizumab-induced renal injury, is a medication used for migraine prophylaxis. It is a Ca^2+^ antagonist that inhibits voltage-dependent slow calcium channels [16]. Flunarizine, a piperazine derivative, also blocks sodium (Na^+^) channels [17]. Flunarizine has been reported to reduce total Ca^2+^ levels and increase antioxidant capacity and ATP levels, and may contribute to the protection against acute renal dysfunction and mitochondrial damage through these mechanisms [18].

Carvacrol, whose potential protective effects against pembrolizumab-induced renal injury are investigated in this study, is a monoterpene phenolic compound used in traditional medicine and as a food additive. Carvacrol has been reported to exhibit antioxidant, antibacterial, antifungal, and anticarcinogenic effects [19]. Carvacrol exerts protective effects against cellular damage by enhancing antioxidant defenses and attenuating inflammation, autophagy, and apoptosis [20]. Therefore, carvacrol is considered a promising compound in the context of nephrotoxicity [21,22].

These findings suggest that flunarizine and carvacrol may be beneficial in the management of pembrolizumab-induced renal toxicity. To the best of our knowledge, no previous study has evaluated these agents comparatively in this context. Flunarizine was considered worthy of investigation in the present study due to its antioxidant, anti-inflammatory, and cytoprotective effects demonstrated in ischemic and oxidative tissue injury models, beyond its classical calcium channel antagonist activity. In particular, flunarizine is known to reduce intracellular calcium overload, membrane instability, and oxidative cellular damage, which are considered important mechanisms involved in pembrolizumab-induced renal injury [23,24]. Therefore, the present study aimed to investigate and compare the potential protective effects of flunarizine and carvacrol against pembrolizumab-induced renal toxicity in rats.

## 2. Materials and Methods

### 2.1. Animals

To ensure homogeneity across experimental groups and minimize potential confounding effects attributable to age- and body weight-related physiological variability, a stringent inclusion criterion was applied during initial screening. Accordingly, 24 male Wistar albino rats confirmed to be 9–10 weeks of age at baseline assessment and falling within a body weight range of 278–287 g were enrolled in the study. The animals employed in this study were sourced from the Experimental Animals Application and Research Center of Erzincan Binali Yıldırım University (Erzincan, Turkey). Following their arrival, the rats were randomly stratified into four experimental groups (*n* = 6 per group) such that comparable mean body weights were achieved across all groups. Before the commencement of experimental procedures, all animals underwent a one-week acclimatization period. Throughout this acclimatization period, the animals were maintained in standard laboratory wire cages measuring 20 cm × 35 cm × 55 cm (floor area: 1925 cm^2^), with six rats housed per cage. Environmental conditions were rigorously controlled throughout the entire duration of the study, encompassing a 12 h light/dark cycle, an ambient temperature of 22 ± 2 °C, and relative humidity maintained within the range of 30–70%. All animals had unrestricted access to standard laboratory chow (Bayramoglu Feed and Flour Industry Inc., Erzurum, Turkey) and tap water throughout the entire experimental period. Prior to the onset of experimental procedures, all rats were confirmed to be in good clinical health, with no observable evidence of systemic or local pathology.

The entirety of animal-related experimental procedures was performed within the laboratory infrastructure of the Experimental Animals Application and Research Center at Erzincan Binali Yıldırım University. All experimental protocols were carried out in full compliance with the European Parliament Directive 2010/63/EU on the protection of animals used for scientific purposes, and adhered to the ARRIVE (Animal Research: Reporting of In Vivo Experiments) reporting guidelines [25]. Considerable care was taken throughout the study to mitigate animal suffering and to keep the number of animals employed at the minimum necessary to fulfill scientific validity requirements.

### 2.2. Chemicals and Reagents

All chemical substances and reagents utilized in the present study were of analytical grade and procured from certified commercial suppliers. Thiopental sodium (Pental Sodium^®^, 0.5 g powder for solution for injection; Catalog No.: 8699508270385) was supplied by Menarini Health and Pharmaceutical Industry Trade Inc. (Istanbul, Marmara Region, Türkiye). Pembrolizumab (Keytruda^®^, 100 mg/4 mL solution for infusion; Catalog No.: 8699636080160; Batch No.: Z012096) was obtained from Merck Sharp & Dohme Pharmaceuticals Ltd. (Istanbul, Marmara Region, Türkiye) through the Oncology Clinic of Erzincan Binali Yıldırım University Mengucek Gazi Training and Research Hospital (Erzincan, Eastern Anatolia Region, Türkiye). Flunarizine (Sibelium^®^, 5 mg tablet; Catalog No.: 8699593015274) was provided by Johnson & Johnson Medical Devices Industry and Trade Ltd. Co. (Istanbul, Marmara Region, Türkiye). Carvacrol was supplied by Sigma-Aldrich Inc. (St. Louis, MO, USA).

### 2.3. Experimental Design and Randomization

In determining the sample size, the principles of the 4R framework (Reduction, Refinement, Replacement, and Responsibility) were strictly adhered to, ensuring that the lowest possible number of animals was employed while maintaining scientific validity and reproducibility [26]. Exclusion criteria were prospectively defined prior to the commencement of any experimental procedures and were organized into two distinct stages: pre-experimental and peri-/post-experimental.

Animals exhibiting abnormal body posture, diminished spontaneous activity, or injuries sustained through aggressive interactions with cage mates were prospectively designated for pre-experimental exclusion. Any animal fulfilling one or more of the aforementioned criteria was prospectively designated for exclusion prior to both randomization and the commencement of experimental treatments. Peri- and post-experimental exclusion criteria encompassed unanticipated mortality prior to the designated experimental endpoints; adverse events arising from anesthesia or drug administration; technical failures including unsuccessful oral gavage or extravasation at the injection site; non-adherence to the predefined treatment schedule or incomplete delivery of study compounds; a decline in body weight surpassing 15–20% of baseline values; clinical manifestations of dehydration or systemic illness; and severe distress reflecting uncontrolled pain or suffering, as evidenced by persistent vocalization or self-injurious behavior. Beyond the aforementioned criteria, any compromise in tissue integrity occurring during specimen collection or processing that had the potential to undermine the accuracy and reliability of biochemical, histopathological, or double immunofluorescence analyses was likewise deemed a valid basis for exclusion.

Rigorous surveillance of all prospectively defined exclusion criteria was maintained throughout the entire intervention period and during the subsequent data evaluation process. None of the animals fulfilled the predefined exclusion criteria at any stage of the experimental period; consequently, all subjects were retained and incorporated into the final statistical analyses. A formal a priori power analysis was not performed in the present study; instead, the sample size was determined based on previously published experimental studies employing comparable methodological designs [18]. Group assignment was carried out by means of a random number table to guarantee impartial and unbiased allocation of animals to their respective experimental groups. To further mitigate potential confounding variables and systematic sources of bias, a unique numerical identification code was assigned to each cage and each individual animal, which was consistently maintained throughout the entirety of the experimental period. All biochemical, histopathological, and double immunofluorescence assessments were conducted by investigators who remained blinded to group allocation throughout the entire analytical process.

### 2.4. Experimental Groups

Four experimental groups were established: the healthy control group (HG, untreated); the pembrolizumab group (PZB, 5 mg/kg, intraperitoneally); the flunarizine-pembrolizumab combination group (FLPZ, 5 mg/kg flunarizine orally + 5 mg/kg pembrolizumab intraperitoneally); and the carvacrol-pembrolizumab combination group (CCPZ, 50 mg/kg carvacrol intraperitoneally + 5 mg/kg pembrolizumab intraperitoneally).

### 2.5. Experimental Procedure

Animals in the FLPZ group (*n* = 6) received flunarizine at a dose of 5 mg/kg via oral gavage, whereas carvacrol was administered intraperitoneally at a dose of 50 mg/kg to animals in the CCPZ group (*n* = 6). Flunarizine was administered orally at a dose of 5 mg/kg, based on prior experimental studies demonstrating its protective effects against drug-induced oxidative tissue injury [27], and carvacrol dissolved in 5% DMSO (dimethyl sulfoxide) [28] was administered intraperitoneally at a dose of 50 mg/kg, based on its established antioxidant and anti-inflammatory efficacy in rat toxicity models [29]. Animals in both the HG (*n* = 6) and PZB (*n* = 6) groups received distilled water via oral gavage as vehicle controls, to replicate the administration procedures applied in the FLPZ and CCPZ groups, respectively. One hour following compound administration, pembrolizumab was intraperitoneally injected at a dose of 5 mg/kg to all animals in the FLPZ, CCPZ, and PZB groups. The pembrolizumab dose of 5 mg/kg was selected based on a previously published experimental study demonstrating its capacity to induce oxidative stress and histopathological tissue damage via intraperitoneal administration in rats [9]. The entire protocol was administered once daily over a consecutive seven-day period. The repeated-dose experimental protocol was designed to establish a reproducible model of cumulative pembrolizumab-induced oxidative renal injury in rats. Upon completion of the treatment regimen, all animals were euthanized via high-dose anesthesia (thiopental sodium, 50 mg/kg), and renal tissues were subsequently harvested for analysis. The biochemical, histopathological, and double immunofluorescence profiles of the collected tissues were systematically compared across all experimental groups.

### 2.6. Biochemical Analyses

#### 2.6.1. Preparation of Samples

Following sacrifice, the kidneys were surgically excised from each animal, and tissue specimens of approximately 50 mg were obtained and weighed. Each specimen was rinsed with ice-cold 0.9% sodium chloride solution to eliminate residual blood and surface contaminants, after which the tissue was minced into small fragments, rapidly snap-frozen in liquid nitrogen, and pulverized to a fine powder using a pre-cooled mortar and pestle. The resulting tissue powder was resuspended in phosphate-buffered saline (PBS, pH 7.4) at a weight-to-volume ratio of 1:10 (*w*/*v*) and subjected to homogenization. The homogenates were briefly vortexed for 10 s and subsequently centrifuged at 10,000× *g* for 20 min at 4 °C. The resulting clarified supernatants were carefully collected and stored at −80 °C until biochemical analyses were performed. All biochemical parameters were normalized to total protein content, with results expressed in units of nmol/mg protein for MDA and tGSH alike.

#### 2.6.2. Determination of MDA, tGSH and Protein Levels in Renal Tissue

Renal tissue malondialdehyde (MDA) and total glutathione (tGSH) levels were quantified using rat-specific enzyme-linked immunosorbent assay (ELISA) kits (MDA: Catalog No. YLA0029RA, Lot No. YLMZYEU; tGSH: Catalog No. YLA0121RA, Lot No. YLMFR73; Shanghai YL Biotech Co., Ltd., Shanghai, China), in accordance with the manufacturer’s instructions. Total protein content was determined using the Bradford assay [30], based on the binding of Coomassie Brilliant Blue G-250 (Sigma-Aldrich Chemie GmbH, Taufkirchen, Bavaria, Germany; Catalog No. 115444) to protein molecules, resulting in a characteristic shift in absorbance. Spectrophotometric measurements were performed at 595 nm.

### 2.7. Histopathological Examination

For the preservation of renal tissue architecture suitable for light microscopic evaluation, all specimens were initially fixed in 10% neutral-buffered formalin. Following fixation, cassette-enclosed tissue samples were subjected to continuous tap water washing for a period of 24 h to ensure complete removal of residual fixative. Dehydration was subsequently accomplished through a graded ethanol series (70%, 90%, and 100%), after which specimens were cleared in xylene and embedded in paraffin. Serial sections of 4–5 µm thickness were obtained using a rotary microtome, mounted onto glass slides, and stained with hematoxylin and eosin (H&E) for routine histopathological evaluation.

Renal tissue sections were obtained from six individual animals per experimental group (*n* = 6), from which consecutive paraffin-embedded sections were subsequently prepared for morphological analysis. Microscopic evaluation was confined to a single randomly selected, non-overlapping field per section at 40× objective magnification, yielding a total of six photomicrographs (one per animal) per group. Imaging was conducted using an Axiolab 5 microscope equipped with an Axiocam 305 color camera and a Colibri 3 LED illumination system (all components: Carl Zeiss Microscopy GmbH, Jena, Thuringia, Germany); image acquisition and processing were performed with ZEISS ZEN 3.1 (Blue Edition) imaging software (Carl Zeiss Microscopy GmbH, Jena, Thuringia, Germany).

Tissue injury was characterized on the basis of one principal histopathological criterion: tubular degeneration. Semi-quantitative assessment was performed according to a four-tiered ordinal scoring system (0: absent, 1: mild, 2: moderate, 3: severe), as outlined in Table 1. To eliminate the possibility of observational bias, all scoring procedures were carried out by a single experienced pathologist who was blinded to group allocation.

### 2.8. Double Immunofluorescence Method

Tissue sections of 5 μm thickness were mounted on poly-L-lysine-coated slides and deparaffinized through xylene and graded alcohol series, followed by washing in phosphate-buffered saline (PBS). For antigen retrieval, sections were treated with citrate buffer (pH 6.0) in a microwave oven (800 W) for two 5 min cycles. After two 10 min washes in PBS, sections were incubated in PBS containing 0.25% gelatin and 0.25% Triton X-100 solution for 10 min to permeabilize cell membranes. Blocking was then performed using 5% bovine serum albumin (BSA) for 1 h at room temperature.

After blocking, sections were incubated overnight at 4 °C with the following primary antibody pairs: mouse monoclonal anti-3,3′-dityrosine (Jaica, Nikken SEIL Co., Ltd., Shizuoka, Shizuoka Prefecture, Japan; Catalog No. MDT-020P) combined with rabbit polyclonal anti-HAVCR1 (Hepatitis A Virus Cellular Receptor 1, Elabscience Biotechnology Co., Ltd., Wuhan, Hubei Province, China; Catalog No. E-AB-93375), and mouse monoclonal anti-COX-1 (anti-Cyclooxygenase-1, Santa Cruz Biotechnology, Inc., Dallas, TX, USA; Catalog No. sc-19998) combined with rabbit polyclonal anti-COX-2 (anti-Cyclooxygenase-2, BT-Lab, Shanghai Korain Biotech Co., Ltd., Shanghai Municipality, China; Catalog No. BT-AP08027), all applied at a dilution of 1:200.

After washing in PBS, sections were incubated for 45 min with a secondary antibody cocktail prepared in 1% BSA at a 1:100 dilution, consisting of goat anti-mouse FITC-conjugated antibody (Jackson ImmunoResearch Laboratories, Inc., West Grove, PA, USA; Catalog No. 115-095-003) and anti-rabbit Alexa Fluor 594-conjugated antibody (Cell Signaling Technology, Inc., Danvers, MA, USA; Catalog No. 8889S), mixed at a 1:1 ratio. Sections were then washed in 10 mM CuSO_4_/50 mM NH_4_Cl solution for 10 min to quench autofluorescence, followed by rinsing in distilled water. DAPI (4,6-diamidino-2-phenylindole) was subsequently applied as a nuclear counterstain.

Immunofluorescence imaging was performed using a Zeiss Axiolab 5 (Carl Zeiss Microscopy GmbH, Jena, Thuringia, Germany) fluorescence microscope equipped with an Axiocam 305 (Carl Zeiss Microscopy GmbH, Jena, Germany) camera and Colibri 3 (Carl Zeiss Microscopy GmbH, Jena, Germany) illumination system. Immunoreactivity for 3,3′-dityrosine and COX-1 was detected in green, whereas immunoreactivity for HAVCR1 and COX-2 was detected in red. Semiquantitative scoring was carried out using ZEISS ZEN 3.1 (Blue Edition) imaging software (Carl Zeiss Microscopy GmbH, Jena, Germany), with immunofluorescence intensity graded on a five-point scale as follows: 0 (absent), 1 (mild), 2 (moderate), 3 (severe), and 4 (very severe).

### 2.9. Statistical Analysis

All statistical procedures were carried out using IBM SPSS Statistics for Windows (Version 27.0, 2020; IBM Corp., Armonk, NY, USA). Biochemical data are presented as mean ± standard error of the mean (SEM), and graphical illustrations of biochemical outcomes were generated using GraphPad Prism (Version 8.0.1, 2018; GraphPad Software, Inc., San Diego, CA, USA). Prior to the application of inferential statistical tests, the distributional characteristics of biochemical data were assessed using the Shapiro–Wilk normality test (Table S1) and Levene’s test for the evaluation of variance homogeneity (Table S2). When both statistical assumptions were fulfilled, intergroup differences were examined using one-way analysis of variance (ANOVA), followed by Tukey’s Honestly Significant Difference (HSD) test as the post hoc procedure for multiple pairwise comparisons (Table S3).

As histopathological injury scores and immunofluorescence intensity values represent ordinal data, non-parametric statistical analysis was performed. Intergroup differences were evaluated using the Kruskal–Wallis test followed by Dunn’s post hoc test for multiple comparisons. Data are presented as median (min–max). A *p*-value < 0.05 was considered statistically significant.

## 3. Results

### 3.1. Biochemical Findings

#### Assessment of MDA and tGSH Levels in Rat Renal Tissue

**Figure 1:**
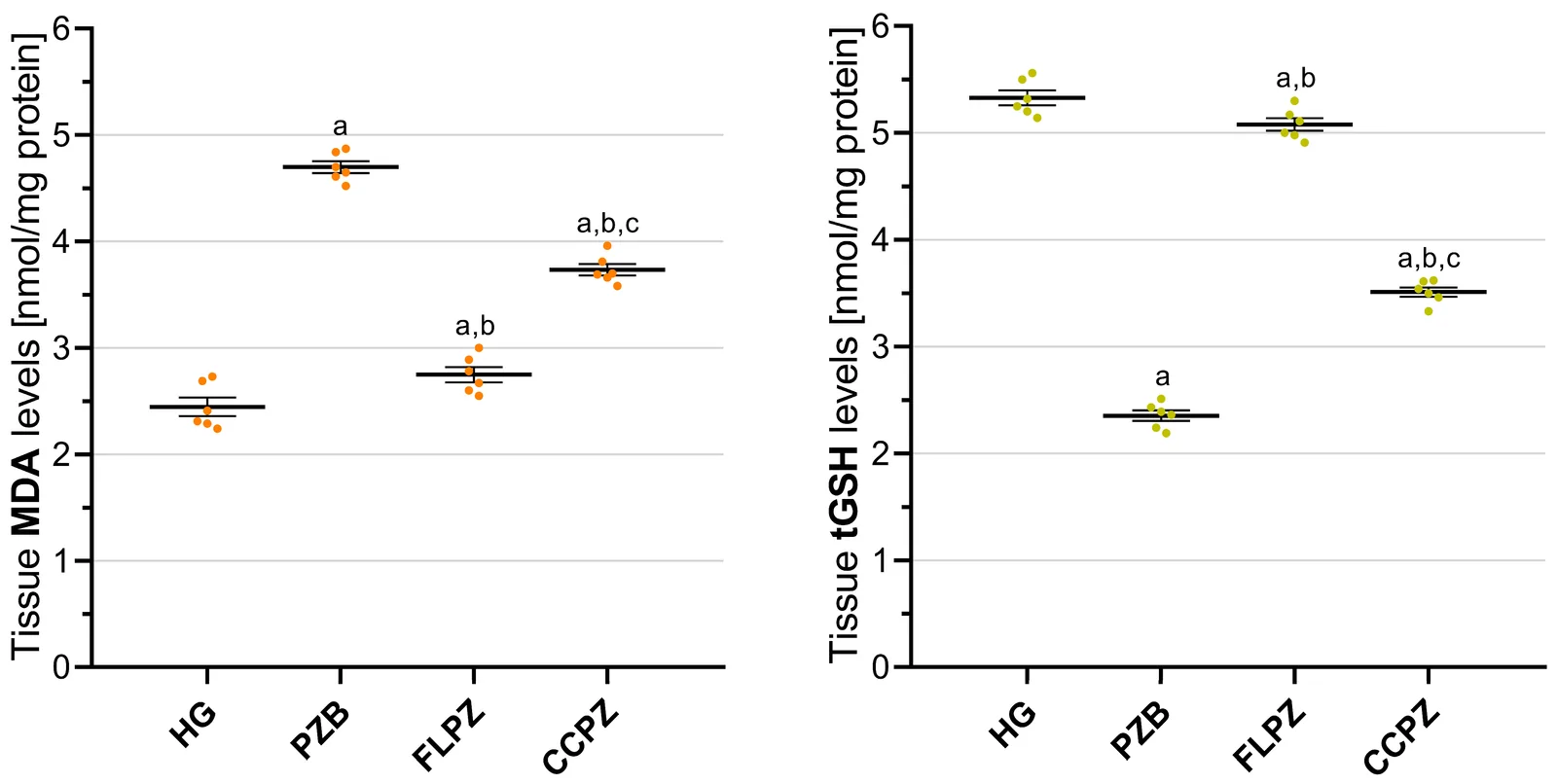
Modulatory effects of flunarizine, carvacrol, and pembrolizumab on MDA and tGSH levels in rat renal tissue. This figure illustrates the effects of pembrolizumab, flunarizine, and carvacrol on oxidative stress parameters in renal tissue. Pembrolizumab significantly increased malondialdehyde (MDA) levels while decreasing total glutathione (tGSH) levels compared with the healthy control group, thereby enhancing oxidative stress. In contrast, both flunarizine and carvacrol significantly attenuated these changes; however, flunarizine was more effective in restoring redox balance. Footnote: Values are presented as mean ± standard error of the mean (SEM). Intergroup statistical comparisons were performed using one-way analysis of variance (ANOVA) in conjunction with Tukey’s Honestly Significant Difference (HSD) post hoc test. Statistical significance is indicated as follows: a, *p* < 0.05 vs. HG; b, *p* < 0.001 vs. PZB; c, *p* < 0.001 vs. FLPZ. Each experimental group consisted of six animals (*n* = 6). Abbreviations: HG, healthy control group; PZB, pembrolizumab-only group; FLPZ, flunarizine and pembrolizumab combination group; CCPZ, carvacrol and pembrolizumab combination group; MDA, malondialdehyde; tGSH, total glutathione.

Renal tissue MDA levels were significantly elevated in the pembrolizumab-treated group (PZB, 4.70 ± 0.06) in comparison with the healthy control group (HG, 2.45 ± 0.09) (*p* < 0.001), as depicted in Figure 1. The concurrent administration of flunarizine (FLPZ, 2.75 ± 0.07) or carvacrol (CCPZ, 3.73 ± 0.05) substantially mitigated the pembrolizumab-induced oxidative burden, with both combination groups exhibiting significantly lower MDA levels relative to the PZB group (FLPZ vs. PZB, *p* < 0.001; CCPZ vs. PZB, *p* < 0.001). Notably, flunarizine pre-treatment conferred a significantly more pronounced inhibitory effect on lipid peroxidation than carvacrol, as reflected by the comparatively lower MDA levels recorded in the FLPZ group versus the CCPZ group (*p* < 0.001). Importantly, MDA levels in the FLPZ group approached those of the healthy control group more closely (FLPZ vs. HG, *p* = 0.024), whereas renal MDA levels in the CCPZ group remained significantly elevated relative to the healthy controls (CCPZ vs. HG, *p* < 0.001). Renal tissue MDA levels were significantly elevated in the pembrolizumab-treated group (PZB, 4.70 ± 0.06) in comparison with the healthy control group (HG, 2.45 ± 0.09) (*p* < 0.001), as depicted in Figure 1. The concurrent administration of flunarizine (FLPZ, 2.75 ± 0.07) or carvacrol (CCPZ, 3.73 ± 0.05) substantially mitigated the pembrolizumab-induced oxidative burden, with both combination groups exhibiting significantly lower MDA levels relative to the PZB group (FLPZ vs. PZB, *p* < 0.001; CCPZ vs. PZB, *p* < 0.001). Notably, flunarizine pre-treatment conferred a significantly more pronounced inhibitory effect on lipid peroxidation than carvacrol, as reflected by the comparatively lower MDA levels recorded in the FLPZ group versus the CCPZ group (*p* < 0.001). Importantly, MDA levels in the FLPZ group approached those of the healthy control group more closely (FLPZ vs. HG, *p* = 0.024), whereas renal MDA levels in the CCPZ group remained significantly elevated relative to the healthy controls (CCPZ vs. HG, *p* < 0.001).

With regard to antioxidant capacity, renal tissue tGSH levels were markedly diminished in the PZB group (2.35 ± 0.05) relative to the HG group (5.33 ± 0.07) (*p* < 0.001), indicative of pembrolizumab-induced antioxidant depletion. Both flunarizine (FLPZ, 5.08 ± 0.06) and carvacrol (CCPZ, 3.51 ± 0.04) pre-treatment significantly restored tGSH levels toward baseline (both *p* < 0.001 vs. PZB). In particular, flunarizine pretreatment yielded a significantly greater degree of antioxidant recovery than carvacrol, as evidenced by the substantially higher tGSH levels observed in the FLPZ group compared with the CCPZ group (*p* < 0.001). Similarly, although tGSH levels in both treatment groups differed significantly from those of the healthy control group, the extent of this difference was substantially smaller in the FLPZ group than in the CCPZ group (FLPZ vs. HG, *p* = 0.023; CCPZ vs. HG, *p* < 0.001) (Figure 1).

### 3.2. Histopathological Findings

**Figure 2:**
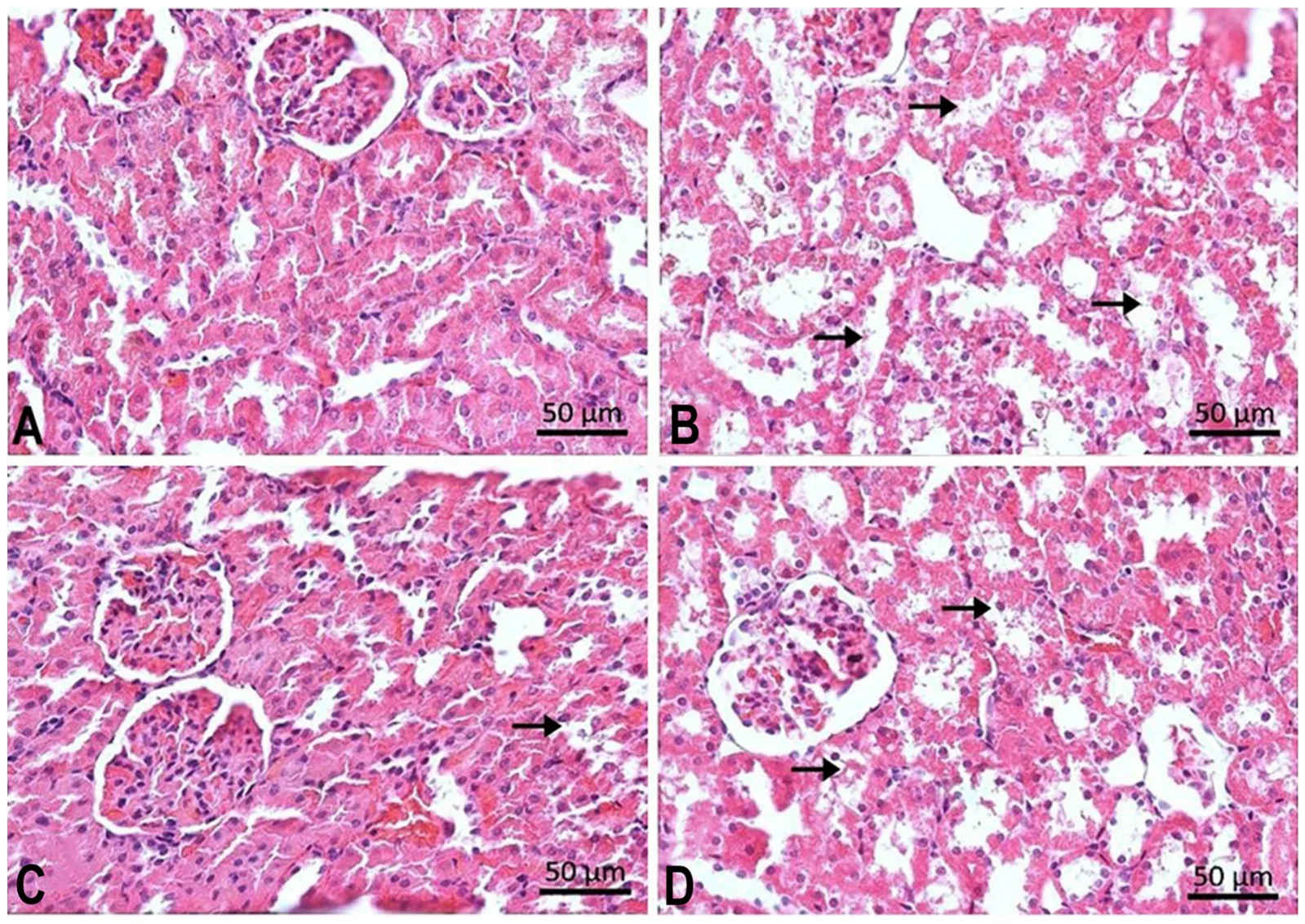
Representative renal histopathological micrographs demonstrating tubular architecture across experimental groups (H&E, ×40). (**A**) HG group: Preserved renal parenchyma with intact tubular morphology, consistent with normal renal histology. (**B**) PZB group: Severe tubular degeneration (arrows), characterized by extensive structural disruption and substantial loss of tubular epithelial integrity. (**C**) FLPZ group: Mild tubular degeneration (arrow), with minimal alterations in tubular epithelial architecture. (**D**) CCPZ group: Moderate tubular degeneration (arrows), with partial disruption of tubular epithelial integrity and evident structural alterations. Tubular injury was evaluated using a semi-quantitative scoring system (0–3) by a blinded pathologist. Data were analyzed using the Kruskal–Wallis test followed by Dunn’s post hoc multiple comparison test. Results are presented as median (min–max), with *p* < 0.05 considered statistically significant.

Semiquantitative histopathological evaluation of renal tissue revealed statistically significant intergroup differences in tubular injury severity (Table 1). The healthy control group (HG) exhibited normal renal histological architecture with intact tubular morphology and no detectable pathological alterations (Figure 2A). Severe tubular degeneration (grade 3) was observed in the pembrolizumab-treated group (PZB) (Figure 2B). The flunarizine pre-treatment group (FLPZ) displayed mild degeneration (grade 1) (Figure 2C). The carvacrol pre-treatment group (CCPZ) showed moderate tubular degeneration (grade 2) (Figure 2D).

### 3.3. Double Immunofluorescence Findings

Double immunofluorescence evaluation of renal tissues revealed that 3,3′-dityrosine and HAVCR1 immunoreactivity was essentially absent in the healthy control group, whereas COX-1 and COX-2 expression was confined to a mild basal level. In contrast, the PZB group exhibited pronounced immunopositivity for both 3,3′-dityrosine and HAVCR1, reaching severe intensity. Comparatively, staining intensity was attenuated in the FLPZ group, where only mild positivity was observed, while the CCPZ group demonstrated a moderate level of immunoreactivity.

A comparable pattern of immunoreactivity was observed for COX-1 and COX-2. COX-1 immunoreactivity was markedly elevated in the PZB group and was graded as very severe, whereas it remained mild in the FLPZ group and severe in the CCPZ group. Similarly, COX-2 expression was very severe in the PZB group, mild-to-moderate in the FLPZ group, and severe in the CCPZ group.

**Figure 3:**
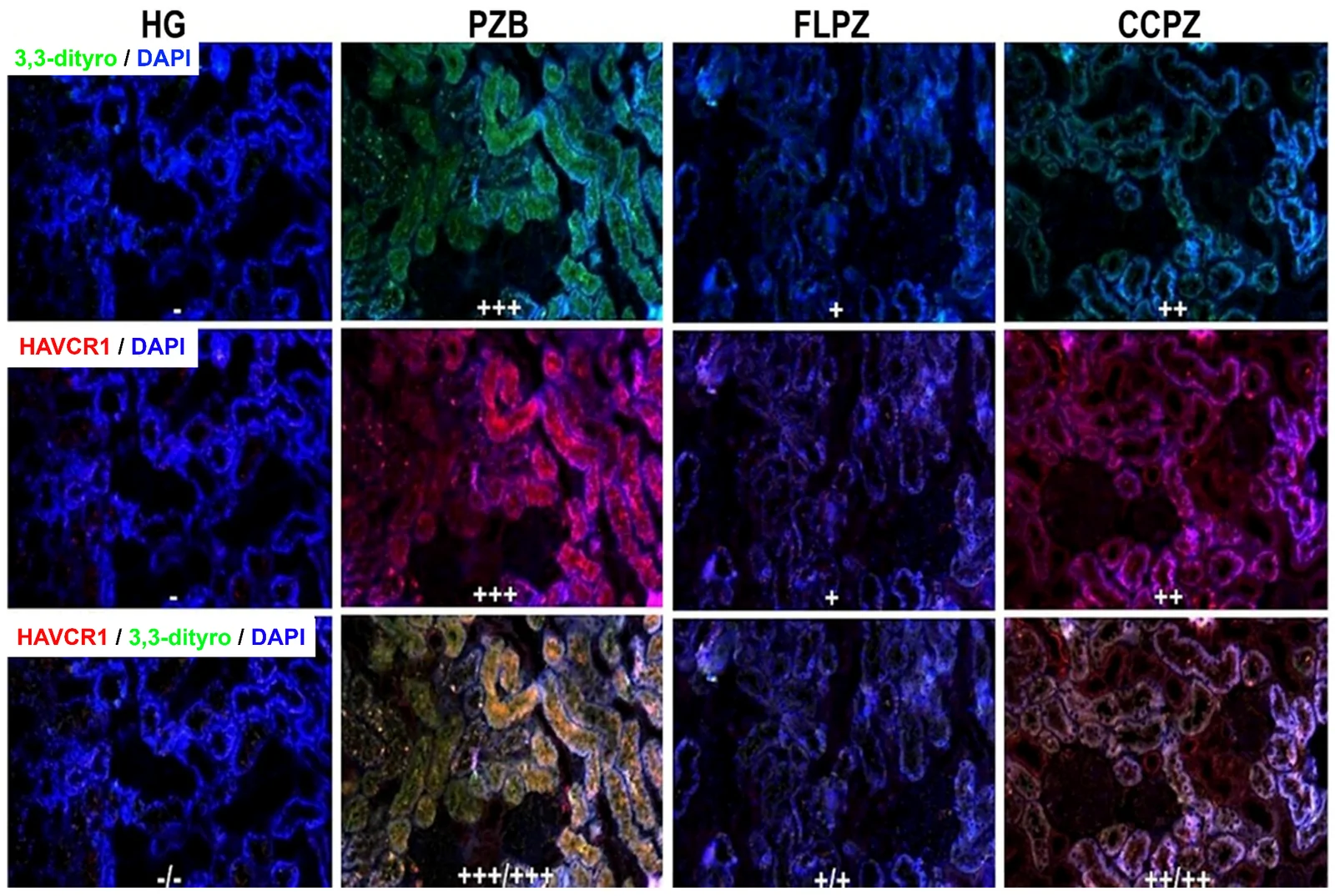
Representative immunofluorescence micrographs illustrating 3,3′-dityrosine and HAVCR1 (Hepatitis A Virus Cellular Receptor 1) immunoreactivities in the HG, PZB, FLPZ, and CCPZ groups. Immunoreactivity was predominantly localized in renal tubular structures. Staining intensity was semiquantitatively graded as absent (-), mild (+), moderate (++), severe (+++), 3,3′-dityrosine immunoreactivity was visualized using FITC, HAVCR1 using Alexa Fluor 594, and nuclei were counterstained with DAPI (4′,6-diamidino-2-phenylindole). In merged images, scores are presented as HAVCR1/3,3′-dityrosine. Magnification: ×40. Scale bar = 50 µm. Data were analyzed using the Kruskal–Wallis test followed by Dunn’s post hoc multiple comparison test. Results are expressed as median (min–max), with *p* < 0.05 considered statistically significant. Abbreviations: HG, healthy control group; PZB, pembrolizumab-only group; FLPZ, flunarizine and pembrolizumab combination group; CCPZ, carvacrol and pembrolizumab combination group.

**Figure 4:**
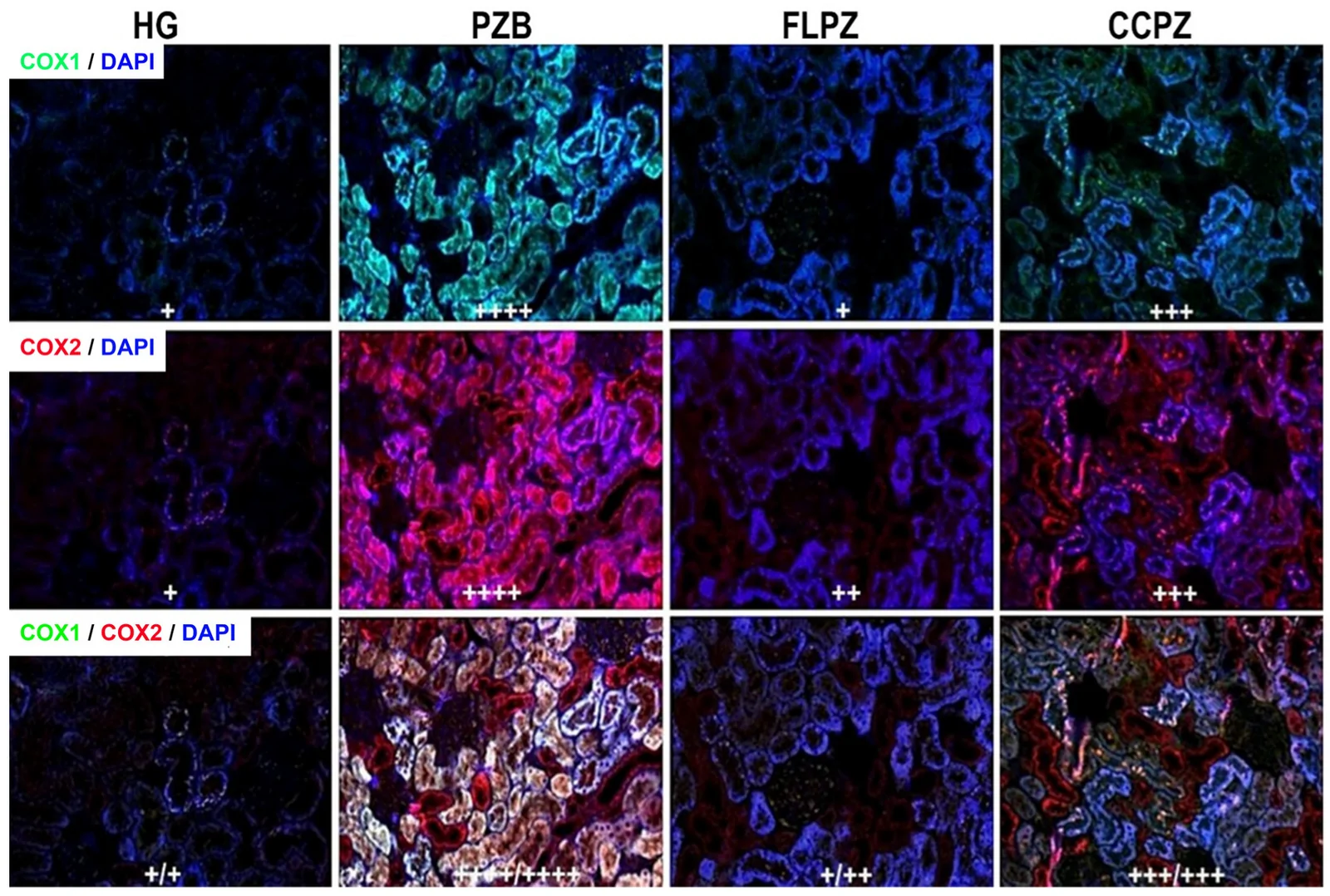
Representative immunofluorescence micrographs showing renal COX-1 (cyclooxygenase-1) and COX-2 (cyclooxygenase-2) immunoreactivity in the HG, PZB, FLPZ, and CCPZ groups. Immunoreactivity was predominantly localized in renal tubular structures. Fluorescence signal intensity was semiquantitatively graded as mild (+), moderate (++), severe (+++), or very severe (++++). COX-1 and COX-2 immunoreactivity were visualized using FITC and Alexa Fluor 594, respectively, and nuclei were counterstained with DAPI (4′,6-diamidino-2-phenylindole). In merged images, scores are presented as COX-1/COX-2. Magnification: ×40. Scale bar = 50 µm. Data were analyzed using the Kruskal–Wallis test followed by Dunn’s post hoc multiple comparison test. Results are expressed as median (min–max), with *p* < 0.05 considered statistically significant. Abbreviations: HG, healthy control group; PZB, pembrolizumab-only group; FLPZ, flunarizine and pembrolizumab combination group; CCPZ, carvacrol and pembrolizumab combination group.

Overall, these data indicate distinct intergroup differences in oxidative stress and inflammatory marker expression across the experimental conditions (Figure 3 and Figure 4, Table 2).

## 4. Discussion

In this study, the protective effects of flunarizine and carvacrol against pembrolizumab-induced nephrotoxicity in rats were systematically assessed using biochemical, histopathological, and double immunofluorescence approaches. Although pembrolizumab confers substantial clinical benefits in oncological settings, it may be associated with a spectrum of adverse effects [5]. Among these, nephrotoxicity has emerged as a clinically relevant complication [8]. Increased oxidative stress, immune cell infiltration, and cytokine release constitute the principal mechanisms underlying this pathogenesis [9]. Our findings revealed that pembrolizumab administration was associated with a significant increase in MDA levels and a marked decrease in tGSH levels in renal tissue. MDA is a toxic oxidant product formed as a result of the oxidation of cell membrane lipids (LPO) by excessively produced ROS. Therefore, the elevation in MDA levels is indicative of increased LPO and oxidative damage to cellular membranes [31]. However, living organisms have developed endogenous antioxidant defense systems to prevent ROS formation or mitigate their harmful effects [32]. GSH is one of the antioxidant defense systems that protects cells from the deleterious effects of oxidative stress [33]. It has also been demonstrated in the literature that pembrolizumab administration may induce oxidative stress, manifested by increases in LPO markers and decreases in antioxidant defense mechanisms such as GSH [9,34]. Although pembrolizumab is a humanized monoclonal antibody targeting the human PD-1 receptor, its effects in rodents are expected to differ. However, our study was designed to mimic downstream pathological processes rather than direct drug–target interactions. The observed increases in oxidative stress and inflammatory markers, together with tubular injury, are consistent with mechanisms reported in immune checkpoint inhibitor–associated nephrotoxicity, particularly acute interstitial nephritis. Therefore, despite inherent interspecies differences, the obtained results provide a mechanistic opportunity to better understand shared pathophysiological pathways involved in pembrolizumab-related renal toxicity in humans.

It has been reported in the literature that pembrolizumab may induce hypercalcemia [12]. Elevated intracellular Ca^2+^ levels are well recognized to be associated with mitochondrial dysfunction, leading to both ATP depletion and ROS overproduction [35]. Flunarizine, investigated in the present study for its potential protective effects against pembrolizumab-induced nephrotoxicity, is a Ca^2+^ channel antagonist [16]. The findings of the present study further support this mechanism. Flunarizine pre-treatment markedly suppressed the pembrolizumab-induced elevation in MDA levels, while the reduction in tGSH levels, a key indicator of the antioxidant defense system, was also substantially mitigated by this treatment. Similarly, flunarizine has been reported to reduce oxidative stress markers and reinforce the antioxidant defense system in various experimental models of tissue injury [36,37]. These findings suggest that flunarizine may exert a protective effect against pembrolizumab-induced oxidative renal damage, and that this protective effect may be associated with the attenuation of intracellular Ca^2+^ influx. Flunarizine has also been shown to both reduce intracellular Ca^2+^ levels and elevate ATP levels [18], and it is therefore proposed that the observed antioxidant effects may be mediated through these mechanisms. However, it should be noted that these mechanistic interpretations remain at a hypothesis level, as Ca^2+^ regulation, mitochondrial function, and ATP levels were not directly assessed in the present study.

Recent studies have demonstrated that natural compounds exert renoprotective effects by suppressing oxidative stress and inflammatory signaling pathways. For example, ginsenoside Rg1 alleviated renal aging by inhibiting caspase-1-mediated inflammation and oxidative stress, while geniposidic acid improved chronic tubulointerstitial nephropathy through regulation of the NF-κB/Nrf2 pathways. These findings provide growing evidence that natural products may serve as promising therapeutic agents for chronic kidney diseases [38,39]. Carvacrol, whose protective effects against renal damage were evaluated in the present study, has been reported to attenuate inflammation, autophagy, and apoptosis, while also exerting antioxidant effects against cellular injury [20]. Our findings support this potential mechanism. Carvacrol pre-treatment was observed to markedly prevent both the pembrolizumab-induced elevation in MDA levels and the reduction in tGSH levels. Forqani et al. reported that elevated MDA levels associated with oxidative damage were inhibited by carvacrol administration [40]. Nouri et al. demonstrated that carvacrol prevented the decline in tGSH levels through its antioxidant properties in an experimentally induced renal injury model [41]. Consistent with the data reported in the literature, our findings suggest that carvacrol may contribute to the attenuation of oxidative stress by suppressing oxidant mechanisms and reinforcing antioxidant defense systems. In addition to experimental findings, clinical studies have also highlighted the renal adverse effects associated with immune checkpoint inhibitors, particularly pembrolizumab-induced kidney injury. Oki et al. [42] reported renal dysfunction following pembrolizumab treatment in patients with non-small cell lung cancer, while Tascon et al. [43], demonstrated subclinical tubular injury associated with immune checkpoint inhibitor therapy using urinary biomarkers These findings emphasize the clinical relevance of developing renoprotective strategies against pembrolizumab-associated nephrotoxicity.

It has been reported that renal tissue damage is among the most frequently observed adverse effects of pembrolizumab in recent years, potentially giving rise to histopathological alterations such as tubular degeneration, interstitial inflammation, and tubular epithelial cell injury [8]. In the present study, tubular degeneration scores were markedly increased in the pembrolizumab-only group. This finding is consistent with previous studies demonstrating that ICIs may induce cellular damage in renal tubular cells through inflammation and oxidative stress [43,44]. In contrast, flunarizine and carvacrol administration was associated with a reduction in tubular degeneration scores. Notably, flunarizine more significantly attenuated pembrolizumab-induced tubular degeneration compared to carvacrol. Evidence in the literature suggests that flunarizine may reduce histopathological alterations and tissue damage in renal tissue through similar mechanisms [18]. Likewise, carvacrol has been reported to limit histopathological damage in renal tissue by virtue of its antioxidant and anti-inflammatory properties [45], through the attenuation of oxidation and inflammation [46].

When the double immunofluorescence findings of the present study are considered together with the biochemical and histopathological results, pembrolizumab administration appears to induce marked oxidative stress and an inflammatory response in renal tissue. The increased expression of 3,3-dityrosine and HAVCR1 in the pembrolizumab-only group is indicative of oxidative damage and inflammation in renal tissue, which is consistent with the existing literature [42]. HAVCR1 is an early and sensitive biomarker of renal injury, the expression of which is upregulated under conditions of renal damage [47]. Similarly, the formation of 3,3′-dityrosine is associated with enhanced oxidative stress [48].

COX-1 and COX-2 expression levels were also found to be elevated in the pembrolizumab group. COX is the rate-limiting enzyme in prostaglandin biosynthesis and exists in two isoforms: COX-1 and COX-2. While COX-1 is constitutively expressed in most cells, COX-2 is generally induced by inflammatory stimuli and plays a role in inflammatory processes [49]. Previous reports have indicated that ICIs may activate the immune system, thereby enhancing inflammatory cytokine production, which in turn may lead to increased COX-2 expression [50]. The elevated COX-1 and COX-2 expression observed in the pembrolizumab group suggests that pembrolizumab-induced immune activation may contribute to inflammation-associated tissue damage. In support of this, an experimental study reported that pembrolizumab administration was accompanied by increases in LPO markers and reductions in antioxidant defense systems, and that antioxidant treatment partially prevented these alterations [9].

The low expression of 3,3′-dityrosine and HAVCR1 observed in the flunarizine group suggests that this agent may exert a protective effect against renal toxicity. It is well established in the literature that COX-1 and COX-2 enzymes play an important role in the regulation of renal function and renal hemodynamics through prostaglandin production [51]. The mild-to-moderate COX expression levels observed in the flunarizine group indicate that flunarizine may suppress inflammatory processes by attenuating intracellular Ca^2+^ influx, which is consistent with previous studies [18]. Although to a lesser extent than flunarizine, the pembrolizumab-induced increases in immunofluorescence immunoreactivity were also partially attenuated in the carvacrol group. The moderate positivity of 3,3′-dityrosine and HAVCR1 is consistent with findings demonstrating that carvacrol protects renal tissue against damage through its antioxidant and anti-inflammatory properties [20,21]. It has further been suggested that, by virtue of these properties, carvacrol may suppress the inflammatory response and reduce COX-2 expression, which plays a role in prostaglandin synthesis [49]. These findings are in line with previous reports [22], suggesting that carvacrol may contribute to the attenuation of oxidative and inflammatory damage developing in renal tissue.

## 5. Conclusions

In the present study, pembrolizumab administration was shown to induce marked oxidative stress, tubular damage, and an inflammatory response in renal tissue. Elevated MDA levels, reduced tGSH levels, and increased expression of 3,3′-dityrosine, HAVCR1, and COX-1/COX-2 collectively substantiate the biochemical and molecular basis of pembrolizumab-induced renal injury. Both flunarizine and carvacrol preserved the oxidant/antioxidant balance and attenuated histopathological damage as well as the elevation in inflammatory markers. Notably, flunarizine demonstrated a more pronounced renoprotective effect compared to carvacrol, as reflected by greater suppression of MDA levels, better preservation of tGSH levels, more effective limitation of tubular damage, and greater attenuation of the increase in double immunofluorescence marker expression. This superior efficacy may be attributable to the ability of flunarizine to inhibit Ca^2+^ influx, thereby preserving mitochondrial integrity. Given the well-established association between elevated intracellular Ca^2+^ levels and mitochondrial dysfunction, ATP depletion, and ROS overproduction, this mechanism may account for the more effective limitation of oxidative stress-induced renal injury observed with flunarizine. In conclusion, both flunarizine and carvacrol may have potential renoprotective effects against pembrolizumab-induced toxicity, with flunarizine showing a comparatively stronger protective profile. Nevertheless, further studies with larger sample sizes and advanced molecular analyses are warranted to translate these findings into clinical practice.

## 6. Limitations and Future Perspectives

The present study has several limitations that should be acknowledged when interpreting the findings. First, the experimental design was conducted exclusively in male Wistar albino rats, and as pembrolizumab is a humanized monoclonal antibody targeting the human PD-1 receptor, its pharmacokinetic and pharmacodynamic profiles in rats differ substantially from those observed in humans; furthermore, the restriction to a single sex precludes generalization of the findings to female subjects, and potential sex-related differences in the renal response to pembrolizumab-induced oxidative injury cannot be excluded. Accordingly, the translational applicability of the present findings to clinical settings warrants careful consideration. Second, the relatively small sample size, determined on the basis of previously published experimental studies rather than a formal a priori power analysis, may limit the statistical power of the study and the generalizability of the results. Third, the study was restricted to single dose levels of flunarizine and carvacrol, administered over a fixed seven-day period; consequently, the dose–response relationships, the optimal therapeutic window, and the long-term renoprotective effects of these agents remain to be established. Fourth, although the biochemical, histopathological, and double immunofluorescence analyses employed in the present study provided a multidimensional characterization of pembrolizumab-induced renal injury, the precise molecular mechanisms underlying the nephroprotective effects of flunarizine and carvacrol were not directly interrogated; in particular, the extent to which flunarizine exerts its protective effects through Ca^2+^/ATP homeostasis and mitochondrial integrity preservation remains to be elucidated through targeted mechanistic studies. Fifth, the present study did not include assessment of serum or urinary biomarkers of renal function, such as blood urea nitrogen and creatinine, which would have provided additional functional correlates to complement the biochemical and histopathological findings. Sixth, the present study did not include flunarizine-only or carvacrol-only control groups; therefore, whether these agents independently affect renal oxidative balance, inflammatory markers, or histological architecture under physiological conditions could not be fully evaluated. This limitation should be considered when interpreting the present findings. Future studies incorporating larger cohorts, both sexes, agent-only control arms, dose-escalation protocols, extended follow-up periods, and mechanistic molecular analyses are warranted to substantiate and extend the present findings toward clinical translation.

## Figures and Tables

**Table 1:** Semiquantitative histopathological assessment of tubular degeneration.

Group	Tubular Degeneration Score (Median [Min–Max])
HG	0 (0–0) ^a^
PZB	3 (2–3) ^d^
FLPZ	1 (1–2) ^b^
CCPZ	2 (2–3) ^c^

Footnote: Histopathological data are expressed as median (minimum–maximum). Tubular degeneration was graded on a four-point scale: 0 = absent, 1 = mild, 2 = moderate, 3 = severe. Overall group comparisons were performed using the Kruskal–Wallis test followed by Dunn’s multiple comparison post hoc test. Different superscript letters indicate statistically significant differences between groups (*p* < 0.05). Each group consisted of six animals (*n* = 6). Abbreviations: HG, healthy control group; PZB, pembrolizumab-only group; FLPZ, flunarizine and pembrolizumab combination group; CCPZ, carvacrol and pembrolizumab combination group.

**Table 2:** Semiquantitative immunofluorescence assessment in renal tissues.

Group	3,3-Dityrosine	HAVCR1	COX-1	COX-2
HG	0 (0–0) ^a^	0 (0–0) ^a^	1 (0–1) ^a^	1 (0–1) ^a^
FLPZ	1 (0–1) ^b^	1 (0–1) ^b^	1 (0–1) ^a^	1 (1–1) ^a^
CCPZ	2 (1–2) ^c^	2 (2–2) ^c^	2 (2–3) ^b^	3 (2–3) ^b^
PZB	3 (2–3) ^d^	3 (2–3) ^d^	3 (3–4) ^c^	3 (3–4) ^c^

Footnote: Immunofluorescence data are expressed as median (minimum–maximum). Semiquantitative staining intensity was graded on a four-point scale: 0 = absent, 1 = mild, 2 = moderate, 3 = severe, and 4 = very severe. Overall group comparisons were performed using the Kruskal–Wallis test followed by Dunn’s multiple comparison post hoc test. Different superscript letters indicate statistically significant differences between groups (*p* < 0.05). Each group consisted of six animals (*n* = 6). Abbreviations: HG, healthy control group; PZB, pembrolizumab-only group; FLPZ, flunarizine and pembrolizumab combination group; CCPZ, carvacrol and pembrolizumab combination group; HAVCR1, Hepatitis A Virus Cellular Receptor 1; COX-1, cyclooxygenase-1; COX-2, cyclooxygenase-2.

## Data Availability

The original contributions presented in this study are included in the article/Supplementary Materials. Further inquiries can be directed to the corresponding author.

## Supplementary Materials

The following supporting information can be downloaded at: https://www.mdpi.com/article/10.3390/biom16060786/s1, Table S1: Distribution normality assessment of biochemical parameters derived from rat renal tissue using the Shapiro–Wilk statistical test; Table S2: Homogeneity of variance assessment for biochemical parameters derived from rat renal tissue using Levene’s statistical test; Table S3: Pairwise post hoc comparison *p*-values for oxidant and antioxidant biomarkers derived from rat renal tissue following administration of flunarizine, carvacrol, and pembrolizumab.
