# Supplementary material for: Targeting Oxidative Stress and Inflammation in Pembrolizumab-Induced Renal Injury: A Comparative Evaluation of the Protective Effects of Flunarizine and Carvacrol in Rats
Source: Biomolecules. 2026 May 27;16(6):786. doi: 10.3390/biom16060786 (PMC13296656; doi:10.3390/biom16060786)
Supplement: Supplementary file 1 [file biomolecules-16-00786-s001.zip › Table S1.pdf]

Table S1. Distribution normality assessment of biochemical parameters derived from rat renal tissue using the Shapiro–Wilk statistical test

		Shapiro–Wilk	Biochemical Variables	
			**MDA**	**tGSH**
Groups	HG	Statistic	0.839	0.920
		df	6	6
		Sig.	0.128	0.502
	PZB	Statistic	0.945	0.960
		df	6	6
		Sig.	0.699	0.822
	FLPZ	Statistic	0.953	0.959
		df	6	6
		Sig.	0.764	0.812
	CCPZ	Statistic	0.926	0.929
		df	6	6
		Sig.	0.551	0.571

Footnote: The Shapiro–Wilk test confirmed that all biochemical parameters exhibited a normal distribution across all experimental groups; accordingly, intergroup statistical comparisons were conducted by means of one-way analysis of variance (ANOVA). Each experimental group consisted of six animals ($n = 6$).

Abbreviations: HG, healthy control group; PZB, pembrolizumab-only group; FLPZ, flunarizine and pembrolizumab combination group; CCPZ, carvacrol and pembrolizumab combination group; MDA, malondialdehyde; tGSH, total glutathione; df, degrees of freedom; Sig., *p*-value.
