# Supplementary material for: Targeting Oxidative Stress and Inflammation in Pembrolizumab-Induced Renal Injury: A Comparative Evaluation of the Protective Effects of Flunarizine and Carvacrol in Rats
Source: Biomolecules. 2026 May 27;16(6):786. doi: 10.3390/biom16060786 (PMC13296656; doi:10.3390/biom16060786)
Supplement: Supplementary file 1 [file biomolecules-16-00786-s001.zip › Table S2.pdf]

Table S2. Homogeneity of variance assessment for biochemical parameters derived from rat renal tissue using Levene's statistical test

	**Biochemical Variables**	
	**MDA**	**tGSH**
Levene's statistic	1.215	0.731
df1	3	3
df2	20	20
Sig.	0.330	0.546

Footnote: Levene's test confirmed the homogeneity of variances for both MDA and tGSH across all experimental groups ($p > 0.05$); consequently, one-way analysis of variance (ANOVA) in conjunction with Tukey's Honestly Significant Difference (HSD) post hoc test was employed for intergroup comparisons. Each experimental group consisted of six animals (n = 6).

Abbreviations: MDA, malondialdehyde; tGSH, total glutathione; df1, numerator degrees of freedom; df2, denominator degrees of freedom; Sig., p-value.
