# Supplementary material for: Targeting Oxidative Stress and Inflammation in Pembrolizumab-Induced Renal Injury: A Comparative Evaluation of the Protective Effects of Flunarizine and Carvacrol in Rats
Source: Biomolecules. 2026 May 27;16(6):786. doi: 10.3390/biom16060786 (PMC13296656; doi:10.3390/biom16060786)
Supplement: Supplementary file 1 [file biomolecules-16-00786-s001.zip › Table S3.pdf]

**Table S3.** Pairwise post hoc comparison *p*-values for oxidant and antioxidant biomarkers derived from rat renal tissue following administration of flunarizine, carvacrol, and pembrolizumab

| | **Post hoc multiple-comparison *p*-values** | |
|---|---|---|
| **Group comparisons** | **MDA** | **tGSH** |
| HG vs. PZB | <0.001 | <0.001 |
| HG vs. FLPZ | 0.024 | 0.023 |
| HG vs. CCPZ | <0.001 | <0.001 |
| PZB vs. FLPZ | <0.001 | <0.001 |
| PZB vs. CCPZ | <0.001 | <0.001 |
| FLPZ vs. CCPZ | <0.001 | <0.001 |
| F-value | 224.109 | 627.984 |
| df (df1 / df2) | 3 / 20 | 3 / 20 |
| *ANOVA* *p*-values | <0.001 | <0.001 |

**Footnote:** Intergroup differences were statistically evaluated by means of one-way analysis of variance (ANOVA), followed by Tukey's Honestly Significant Difference (HSD) post hoc test for multiple pairwise comparisons. Each experimental group comprised six animals ($n$ = 6).
**Abbreviations**: HG, healthy control group; PZB, pembrolizumab-only group; FLPZ, flunarizine and pembrolizumab combination group; CCPZ, carvacrol and pembrolizumab combination group; MDA, malondialdehyde; tGSH, total glutathione; df, degrees of freedom; df1, numerator degrees of freedom; df2, denominator degrees of freedom.
